# Development of Specific Aspects of Spirituality during a 6-Month Intensive Yoga Practice

**DOI:** 10.1155/2012/981523

**Published:** 2012-07-18

**Authors:** Arndt Büssing, Anemone Hedtstück, Sat Bir S. Khalsa, Thomas Ostermann, Peter Heusser

**Affiliations:** ^1^Center of Integrative Medicine, Faculty of Health, Witten/Herdecke University, Gerhard-Kienle-Weg 4, 58313 Herdecke, Germany; ^2^Division of Sleep Medicine, Brigham and Women's Hospital, Harvard Medical School, Boston, MA 02115, USA; ^3^Theory in Medicine, Integrative and Anthroposophic Medicine, Faculty of Health, Witten/Herdecke University, Gerhard-Kienle-Weg 4, 58313 Herdecke, Germany

## Abstract

The majority of research on yoga focuses on its psychophysiological and therapeutic benefits, while the spiritual aspects are rarely addressed. Changes of specific aspects of spirituality were thus investigated among 160 individuals (91% women, mean age 40.9 ± 8.3 years; 57% Christians) starting a 2-year yoga teacher training. We used standardized questionnaires to measure aspects of spirituality (ASP), mindfulness (FMI—Freiburg Mindfulness Inventory), life satisfaction (BMLSS—Brief Multidimensional Life Satisfaction Scale), and positive mood (*lightheartedness/relief*). At the start of the course, scores of the respective ASP subscales for *search for insight/wisdom, transcendence conviction*, and *conscious interactions/compassion* were high, while those for *religious orientation* were low. Within the 6 month observation period, both *conscious interactions/compassion* (effect size, Cohen's *d* = .33), *Religious orientation* (*d* = .21), *Lightheartedness/Relief* (*d* = .75) and mindfulness (*d* = .53) increased significantly. Particularly non-religious/non-spiritual individuals showed moderate effects for an increase of *conscious interactions/compassion*. The results from this study suggest that an intensive yoga practice (1) may significantly increase specific aspects of practitioners' spirituality, mindfulness, and mood, (2) that these changes are dependent in part on their original spiritual/religious self-perception, and (3) that there are strong correlations amongst these constructs (i.e., *conscious interactions/compassion*, and mindfulness).

## 1. Introduction

 Historically, yoga is a contemplative discipline originating on the Indian subcontinent that incorporates both physical and mental practices with the goal of achieving unitive states of consciousness and spiritual advancement. Modern yoga practices are associated with numerous different schools or types of yoga (i.e., Iyengar, Viniyoga, and Sivananda), each with distinct priorities in terms of their balance of spiritual and physical practices [1]. Components of yoga practice include distinct postures (asanas), control of breath (pranayama), deep relaxation techniques, and the practice of meditation to cultivate awareness and mindfulness. Yoga practices have become popular, in large part, due to their ability to produce psychophysiological changes that reduce the activity of the stress response systems and enhance self-regulation, resilience, mood, well-being, and quality of life [2]. These psychophysiological benefits have contributed to the rationale for the use of yoga as a therapeutic intervention in a variety of physical and psychological conditions [3] including depression [4, 5], chronic pain conditions [6, 7], low back pain [8] and arthritis [9], and several other conditions [10].

Although yoga is a practice that does not require adoption of religious beliefs or dogma, its practices are aimed at the experience of contemplative states of consciousness and spirituality. Yoga can be rightly categorized as a practicaly applied philosophy within the philosophical discipline of mysticism, whose primary tenet is the experience of a transcendent, unitive state of consciousness [11]. However, to date, the majority of research on yoga has been focused on its psychophysiological and therapeutic benefits, and there are relatively fewer studies which address the spiritual aspects of yoga practices.

An early study divided undergraduate meditators into separate categories of meditation experience (transcendental meditation or various Buddhist styles) as nonmeditators, beginners, and short-term and long-term meditators [12]. Subjects were administered the Shor Personal Experience Questionnaire assessing personality variables which predict hypnotizability and reflect state of consciousness and experience, and the Tellegen Absorption Scale that correlates consistently with hypnotizability and defines absorption as a disposition for having episodes of total attention and immersion in an attentional object. Scores on these questionnaires showed significant increases with meditation experience suggesting that meditation practice induces long-term changes in state of consciousness and experience [12].

A study of members of a yoga ashram revealed that they showed statistically greater psychological characteristics of state of consciousness, and after-effects and interpretation of positive life experiences than those experienced by a control group of non-ashram residents [13]. Ashram respondents showed a higher percentage of positive responses on a number of indices, including “Felt a personality change,” “Experience resulted in change in life,” “Experience of oneness,” and “In touch with divine or spiritual” [13]. These data would support the notion that yoga practice may enhance transformational processes, including spiritual, transcendent states.

Monk-Turner and Turner [14] investigated mental wellness in yoga practitioners and college students, and found that the students reported more mental wellness than the yoga practitioners. Moreover, four of five measures of spiritual health differed significantly between yoga practitioners and college students; that is, more yoga practitioners felt “that they expressed their spirituality appropriately and in a healthy way,” “recognized the positive contribution faith could make to the quality of life,” and “had a positive outlook on life”, while more college students “rarely undertook new experiences to enhance their spiritual health” [14]. Although one may criticize the value of these items and whether the samples are comparable, the data are nevertheless important to generate hypotheses for future studies.

In 28 Japanese cancer patients participating in two sessions of mindfulness-based meditation therapy (which included yoga practices), Ando et al. [15] reported a marginal, nonsignificant increase of spiritual well-being. Nevertheless, spiritual well-being was related to anxiety depression, and pain [15]. A recent prospective study of undergraduates with mild to moderate depression, anxiety, or stress reported improvements in a number of mood and stress measures, but not in spiritual well-being after a 7-week yoga intervention [16]. However, the use of relatively naïve subjects over a short time frame in subjects with mood and/or stress impairment may account for this, and it may require longer practice to achieve measurable improvements in spirituality. On the other hand, studies of yoga interventions in cancer patients have reported improvements in measures of spirituality relative to controls [16, 17]. Particularly the meaning/peace component of spiritual wellbeing (FACIT-Sp) increased within 10 weeks in yoga groups (but not their emotional, social, or functional well-being), while in the respective control group these variables would not change or even decrease [17]. This would indicate a specific effect of yoga with respect to spiritual wellbeing.

MacDonald and Friedman [18] provided information on putatively relevant measures of spiritual and transpersonal constructs which could be used in yoga research. They stated that it was their intention “that the information presented in this article serves as a catalyst for new avenues of spiritual and transpersonal research in the area of yoga studies”. Whether or not this paper has encouraged studies on this topic remains unclear.

We thus intended to investigate whether and how specific aspects of spirituality may change within a 6-month intensive yoga practice. To address this question we chose individuals who were already practicing yoga and decided to start a yoga teacher training, which would subject them to a greater intensity, frequency, and duration of practice. As a standardized measure of specific aspects of spirituality, we used an instrument which addresses secular aspects of spirituality (i.e., conscious interaction, compassion, and existential issues), religious issues, and cognitive transcendence convictions. This *Aspects of Spirituality* (ASP) Questionnaire differentiates and quantifies cognitive, emotional, intentional, and action-oriented matters [19, 20].

## 2. Materials and Methods

### 2.1. Participants

In an anonymous prospective pre-post study, we enrolled 191 individuals at the beginning of a 2-year yoga teacher training in Yoga Vidya Centers of Bad Meinberg and Westerwald (Germany) who gave informed consent to participate (90% of all participants).

At the first training weekend in these centers a Yoga Vidya cooperator gave a short instruction of the study to the participants. All provided informed consent to participate prior to completing the questionnaire (starting in March 2010) which neither asked for name nor initials nor address. The study was approved by the ethical commission of our institution (no. 23/2010).

We acquired outcome measures at the start of the intensified training (T1), 3 months later (T2) and again 6 months later (T3). Finally, 160 individuals identified by self-given code names provided sufficient data quality with at least two time points (75.5% of all participants). We excluded individuals because of nonidentification, insufficient answering, or missing follow-up questionnaires.

### 2.2. Practices

During the first 6 months of the 2-year standardized Yoga Vidya yoga teacher-training program, the trainees learned and practiced specific postures (asanas); breathing practices (pranayama), relaxation and meditation practices, and specific mantras. Moreover, they were also provided with instructions in the cultivation and development of positive qualities and attitudes based on the classical yoga teachings, and given lectures on yoga philosophy, and so forth. Trainees were also encouraged to read recommended books on yoga practices at home.

Specifically, the basic schedule included a weekly 3 h evening tutorial in small groups at individual subcenters all over Germany. Within each session, they were given special and sometimes personal exercises to practice during the week. Additionally, participants attended intensive training weekends in the main centers about every 2 months. The first one was after 3 weeks after the beginning in the individual subcenter. The surveys at T1, T2, and T3 took part in the first 3 sessions in the main centers.

Furthermore, participants were advised to read and study the Yoga Vidya teacher manual and practice at least 1 h daily at home, including asana, pranayama, meditation, relaxation, and/or mantras. Also, they were encouraged to follow the yogic lifestyle as far as possible in their daily life, including adoption of a vegetarian diet, renunciation from drugs (including caffeine), and ethical behavior as described in Patanjali's Yoga Sutras.

Subjects within each tutorial or intensive training week-end were a mixture of theory of yoga and its lifestyle, asana, pranayama, relaxation, mantra, and meditation. Each subject was instructed in both theoretical and practical aspects of yoga practice. The focus of the tutorials was on achieving proper practice for each participant, self-development, and development of skills for yoga instruction as a teacher.

### 2.3. Measures

#### 2.3.1. Aspects of Spirituality

To measure a wide variety of important aspects of spirituality beyond conventional conceptual boundaries, we used an instrument which was developed on the basis of the answers of expert representatives of various spiritual orientations who were asked to report on which aspects of spirituality are relevant to them (i.e., Catholics, Protestants, members of the Anthroposophic “Christengemeinschaft", Bahá'í, Muslims, Jews, Buddhists, and atheists) [19]. The identified motifs we condensed to 40 items of the original ASP questionnaire [18]. For this analysis, we used the 25-item ASP 2.1 [20] which differentiates (1) *religious orientation: prayer/trust in God* (religious views; 9 items, Cronbach's alpha = .93), (2) *search for insight/wisdom *(philosophical/existentialist views; 7 items, alpha = .88), (3) *conscious interactions/compassion* (conscious interactions with other, self, environment, compassion, generosity; 5 items, alpha = .83), and (4) *transcendence conviction* (beliefs in rebirth, existence of higher powers and beings, soul has his origin in a higher dimension, and man is a spiritual being; 4 Items, alpha = 85). The specific term *God* was used only once. All items were scored on a 5-point scale from disagreement to agreement, that is, 0: disagree strongly; 1: disagree; 2: neutral; 3: agree somewhat; 4: agree strongly). The scores refer to a 100% level, where 4: agree strongly = 100%.

#### 2.3.2. Spiritual/Religious Self-Categorization

According to their responses to the SpREUK items f2.6 (“To my mind I am a religious individual” = R) and f1.1 (“To my mind I am a spiritual individual” = S), the practitioners were categorized as religious but not spiritual (R+S−), as not religious but spiritual (R−S+), as both religious and spiritual (R+S+), or as neither religious nor spiritual (R−S−). To avoid internal conflicts, we did not provide information how a religious or a spiritual individual should be defined; nevertheless, previous studies have shown that this self-categorization is consistent with patients' spiritual/religious convictions [21, 22] and their engagement in specific forms of spiritual/religious practices [23, 24].

#### 2.3.3. Mindfulness

Mindfulness was measured with the Freiburg Mindfulness Inventory (FMI) [25]. For this study, we used the 14-item short version [26] which was found to be semantically robust and psychometrically stable (Cronbach's alpha =.83). “Examples of items are “watch my feelings without getting lost in them”, “open to the experience of the present moment”, “feel connected to my experience in the here-and-now”, “in difficult situations, I can pause without immediately reacting”, “experience moments of inner peace and ease, even when things get hectic and stressful” and so forth”. All items were scored on a 4-point scale (0: rarely; 1: occasionally; 2: fairly often; 3: almost always). FMI scores are given as sum scores.

#### 2.3.4. Life Satisfaction

Life satisfaction was measured using the *Brief Multidimensional Life Satisfaction Scale* (BMLSS) [27] which uses items of Huebner's “Brief Multidimensional Students” Life Satisfaction Scale [28, 29] and was tested among adults [23]. The eight items of the BMLSS address intrinsic (*Myself*, *Life in general*), social (*friendships*, *family life*), external (*School situation*, *Where I live*) and prospective (*Financial situation*, *Future prospects*) dimensions. The internal consistency of the instrument was good (Cronbach's alpha =.87) [27]. Each item was introduced by the phrase “I would describe my level of satisfaction as …”, and scored on a 7-point scale from dissatisfaction to satisfaction (0: terrible; 1: unhappy; 2: mostly dissatisfied; 3: mixed (about equally satisfied and dissatisfied); 4: mostly satisfied; 5: pleased; 6: delighted). The BMLSS sum score refers to a 100% level (delighted).

#### 2.3.5. Lightheartedness/Relief

The *lightheartedness/relief *scale  was taken from the German language ERG (emotional and physical reactions) questionnaire [30], asking for specific perceptions, reactions, and feelings in terms of patient's dealing with illness. The intention was to assess the association of distinct (emotional and behavioral) attitudes with a revival of vitality and zest of life, that is, positive internal attitudes such as easiness and subsequent openness to external contacts (“*social interest/contacts*”). These attitudes were operationalized in the context of an increasing positive health/well-being instead of a decrease of functional and emotional health affections and deficiencies. The primary scale to address this “external warming” has 9 items, and a 2-factorial structure with satisfying internal consistency coefficients, that is, Lightheartedness/Relief (LHR; Cronbach's *alpha* = .74) and Social Interest/Contact (Cronbach's *alpha* = .79) [30]. For this analysis we focused on the 5-item subscale LHR because in a previous study it showed significant contrary qualities to psychical exhaustion (correlation *r* = −.49) and disturbed sleep regeneration (*r* = −.53), correlated moderately with *social interest/contacts* (*r* = .43) [30], strongly associated with positive mood, and moderately associated with mindfulness, mental health, and life satisfaction [31]. The items address feelings such as “felt (internally) deeply relieved,” “specific affairs succeeded better and better,” “movements are easy and fluid,” “filled with bright happiness,” and a reverse statement “felt empty within”. They were scored on a 5-point scale, from disagreement to agreement (0: does not apply at all; 1: does not truly apply; 2: I cannot decide/cannot say; 3: applies quite a bit; and 4: applies very much). The final scores referred to a 100% level (transformed scale score).

### 2.4. Statistical Analyses

All statistical analyses were performed with SPSS 17.0/20.0 for Windows (SPSS GmbH Software, Munich). We considered a level of *P* < 0.05 as statistically significant. To assess the pre-post effects, we compared data from the start (T1), after 3 months (T2), and after 6 months (T3), using the Wilcoxon signed rank test (T1 versus T2, T1 versus T3, and T3 versus T2), and to assess differences across the time (T1 ≤ T2 ≤ T3) the Friedman test. Effect sizes were expressed as Cohen's d [32] using T1 and T3 data. According to Cohen [32] and Wolff [33], we judged effect sizes >0.8 as indicators of large effects and effect sizes from 0.5 to 0.8 as indicators of moderate effects.

## 3. Results

### 3.1. Individuals

Among the 191 enrolled individuals (90% of all participants), 160 individuals provided sufficient data quality with at least two time points (75.5% of all participants). Their mean age was 40.9 ± 8.3 years; 11% were <31 years of age, 37% between 31 and 40 years, 36% between 41 and 50 years, and 16% >51 years. Their mean duration of yoga practice was 39 ± 53 months; range: 2 to 384 months).

Most were women (91%), were living with a partner (67%), and had a high school education (55%). Sixty-eight percent stated to be healthy, 9% had psychological/psychiatric disorders, 4% chronic pain diseases, 2% cancer, and 17% other chronic conditions (Table 1).

A large fraction (38%) stated that they have no religious orientation, 57% had a Christian denomination, and 5% other. Among them, 29% regarded themselves as religious and spiritual (R+S+), 5% as religious but not spiritual (R+S−), 43% as not religious but spiritual (R−S+), and 23% as neither religious nor spiritual (R−S−) (Table 1).

As compared to reference data [26], their life satisfaction and *social interest/contacts* scores were high, while *lightheartedness/relief* scores were in the medium range with respect to the 0–100 scale (Table 2).

### 3.2. Aspects of Spirituality

At the start of the course, scores for *Search for insight/wisdom*, *Transcendence conviction and Conscious interactions/compassion were high*,* while Religious orientation*: *prayer/trust in God *were very *weak* (Table 2).

*Religious orientation* was strongly associated with *Transcendence conviction*, *Search for insight/wisdom *and *Conscious interactions/compassion *(Table 3). *Transcendence conviction *was also strongly associated with *Search for insight*/*wisdom*. *Conscious interactions/compassion *was moderately correlated with life satisfaction, *lightheartedness/relief* and *social interest/contacts*, and strongly with mindfulness. Similarly, *Religious orientation* was moderately associated with mindfulness, life satisfaction and *lightheartedness/relief* (Table 3).

Within the 6-month observation period (Table 2), both *Conscious interactions/compassion* (*d* = .33) and *Religious orientation* (*d* = .21) increased significantly, while *Transcendence conviction* did not change significantly over time (*d* = .02), and *Search for Insight/wisdom* showed a marginal change (*d* = .10). In parallel, *lightheartedness/relief *(*d* = .75) and mindfulness (*d* = .53) increased significantly within the 6-month observation period, while life satisfaction increased marginally (*d* = .14) and *social Interest/contacts *remained unchanged (*d* = .09) (Table 2).

Detailed analyses revealed that the underlying SpR attitude (self-categorization) may have had an impact on which aspects of spirituality develop over the course time.  Table 4 indicates that particularly R−S− individuals showed a moderate effect with respect to *Conscious interactions/compassion. *Moreover, both R+S+ and R−S− individuals showed strong effect sizes for mindfulness and *lightheartedness/relief*, while their R+S− counterparts showed only weak effect sizes. While individuals with chronic conditions had stronger effects with respect to *Religious orientation*, *Search for insight/wisdom *(which decreased)  and *lightheartedness/relief* than their healthy counterparts, the effect sizes of the other variables did not differ.

## 4. Discussion

We have shown that among yoga practitioners, compared to their *Religious orientation*, scores on the scales for *Search for insight/wisdom* and *Transcendence conviction* were high even before the start of their intensive practice; nevertheless, compared to reference values of a population of the same age [30], these data are slightly lower, but within the range. Nevertheless, it was obvious that the yoga practitioners' *Transcendence conviction* was strongly related with their *Search for insight/wisdom *and  also conventional *Religious orientation*. This suggests that constructs such as belief in rebirth and existence of higher powers and beings, and so forth, religious beliefs including praying, trust in God who guides and shelters, and the attempt to “express the Divine in the creation” are not necessarily independent of yoga practitioners' spirituality. Moreover, in contrast to the reference population, where *Religious orientation* was just moderately associated with *Search for insight/wisdom* and *Conscious interactions/compassion* [19], these associations were strong in the yoga practitioners. However, most of these yoga practitioners do not regard themselves as religious (66%), but at least spiritual. One could argue that their *Religious orientation* is defined by specific attitudes and private practices which are compatible with, or possibly enhanced by, their yoga practice. On the other hand, some practitioners stated that while they have turned away from institutionalized religion years before, they have now re-discovered their relationship to their Christian traditions and have become more familiar with the underlying beliefs and practices—via their yoga practice (personal communications). In fact, the intense yoga practice appears to be associated with weak increases of both *Conscious interactions *(with others, self and environment)*  * and *Religious orientation*.

The fact that *Conscious interactions/compassion* increased within the observation period would mean that they may have developed a heightened awareness of their actions and their implications, suggesting an increase in moral development. Similarly, their mindfulness scores increased strongly. Although one cannot exclude the possibility that these increases could be also attributed to their positive (and socially desired) expectation that the yoga practice should result in mindful interactions with others, self, and environment, this would not necessarily explain the strong increase of *lightheartedness/relief. *This scale addresses feelings such as “felt (internally) deeply relieved,” “specific affairs succeeded better and better,” “movements are easy and lope,” “filled with bright happiness,” and a reverse statement “felt empty within.” One may expect that an intense yoga practice would foster these experiences in terms of a physical and psychoemotional development. But it did not result in a further increase of life satisfaction (the increase was just marginal) or *social interest/contacts*.

Of interests are additional analyses which indicate that R−S− had a stronger increase *of Conscious interactions* and *Religious orientation* when compared to their R+S+ or R−S+ counterparts. This might suggest that because of their primarily low interest in spiritual issues and thus low practical experience, they seemed to benefit from the spiritual component of the yoga practice. In contrast, both R+S+ and R−S− individuals showed strong effect sizes for mindfulness and *lightheartedness/relief*, while their R+S− counterparts showed just weak effects. We have so far no sound explanations for this observation. Further analyses revealed that the individuals with chronic conditions had a stronger positive development of their *Religious orientation* and *lightheartedness/relief* than their healthy counterparts. This might suggest that these individuals who have experienced chronic diseases are more open or even in need of a religious resource to assist in coping which seems to be provided by the yoga practice. In fact, there is an increasing amount of evidence that spirituality/religiosity can be an important coping strategy related to improved medical outcomes (reviewed by [34]).

One limitation of this study is that we had no control group. However, it is unlikely that spiritual characteristics and mindfulness would have changed in a passive control group within a six-month observation period by chance. Therefore, we have defined cohorts of practitioners that differ with respect to their “inner involvement” (ICPH) with the (yoga) practices [35] which has allowed us to assess the practitioners' courses of variables addressing spirituality and mental health. The respective findings will be addressed in a different paper.

## 5. Conclusion

The results from this study suggest (1) that an intensive yoga practice may significantly increase specific aspects of practitioners' spirituality, mindfulness, and mood, (2) that these changes are dependent in part on their original spiritual/religious self-perception, and (3) that there are strong correlations amongst these constructs. Future studies are needed to further evaluate the potentially important role of yoga practices in enhancing positive psychology. These studies are currently underway. Yoga may be a practice that could effectively contribute to manifesting the World Health Organization's definition of health as a “state of complete physical, mental, and social well-being, and not merely the absence of disease or infirmity” [36], but could extend beyond this to also include the cultivation of spiritual wellbeing.

## Figures and Tables

**Table 1:** Demographic data of yoga practitioners.

Gender (%)	
Women	91
Men	9

Mean age (years)	40.9 ± 8.3

Family status (%)	
Married	50
Living with partner, not married	17
Alone	20
Divorced	13

Educational level (%)	
Secondary (Hauptschule)	8
Junior high school (Realschule)	27
High school (Gymnasium)	55
Other	11

Religious denomination (%)	
Christian	57
Other	5
None	38

Spiritual self-categorization^∗^ (%)	
R+S+	29
R+S−	5
R−S+	43
R−S−	23

Chronic diseases (%)	32
Psychic disorders	9
Chronic pain diseases	4
Cancer	2
Other chronic affections/disorders	17

Mean duration of yoga practice (months)	39 ± 53

^∗^R: religious; S: spiritual.

**Table 2:** Course of tested variables.

	Mean ± SD (Wilcoxon)			*P* value	Effect size *d*
	T1	T2	T3	(Friedman)	T1 : T3
Religious orientation	61.0 ± 23.3	62.0 ± 24.4	65.9 ± 23.4^2∗∗3∗∗∗^	<0.0001	0.21
Insight/wisdom	82.6 ± 14.3	80.0 ± 15.9	81.3 ± 14.7	0.009	0.10
Conscious interactions/compassion	75.2 ± 13.8	77.3 ± 13.6^1∗^	79.6 ± 12.6^2∗∗∗3∗∗^	0.009	0.33
Transcendence conviction	79.9 ± 17.5	78.9 ± 20.1	81.9 ± 28.9	0.399	0.02

Mindfulness	1.78 ± 0.35	1.86 ± 0.35^1∗∗^	1.98 ± 0.37^2∗∗∗3∗∗∗^	<0.0001	**0.53**
Life satisfaction	75.4 ± 13.8	77.0 ± 14.5^1∗∗^	77.4 ± 13.8^2∗^	0.016	0.14
Lightheartedness/relief	55.7 ± 11.9	58.3 ± 14.4^1∗^	66.3 ± 17.2^2∗∗∗3∗∗∗^	<0.0001	**0.75**
Social interest/contacts	71.1 ± 17.9	71.1 ± 18.0	72.6 ± 17.0	0.274	0.09

^1^T1 : T2; ^2^T1 : T3; ^3^T2 : T3 ^∗∗∗^ *P* < 0.001; ^∗∗^ *P* < 0.01; ^∗^ *P* < 0.05 (Wilcoxon; 2-tailed).

**Table 3:** Correlation analyses (T1).

	Religious orientation	Insight/wisdom	Conscious interactions/compassion	Transcendence conviction
Religious orientation	1	**.505** ^ ∗∗^	**.576** ^ ∗∗^	**.708** ^ ∗∗^
Insight/wisdom		1	.447^∗∗^	**.629** ^ ∗∗^
Conscious interactions/compassion			1	.448^∗∗^
Transcendence conviction				1

Mindfulness	.482^∗∗^	.298^∗∗^	**.542** ^ ∗∗^	.308^∗∗^
Life satisfaction	.349^∗∗^	.042	.330^∗∗^	.143
Lightheartedness/relief	.313^∗∗^	.136	.349^∗∗^	.179
Social interest/contacts	.175	.196	.343^∗∗^	.165

Strong correlations were highlighted (bold).

^∗∗^ *P* < 0.01 (Pearson).

**Table 4:** Effect sizes with respect to SpR self-categorization and chronic conditions versus healthy.

T1 : T3 effect size *d * (mean difference)	Religious orientation	Insight/wisdom	Conscious interactions/compassion	Transcendence conviction	Mindfulness	Lighthearted-ness/relief
R+S+ (29%)	0.28 (4.20)	0.03 (0.30)	0.20 (2.50)	0.09 (1.20)	**0.87** (0.27)	**1.08** (14.20)
R−S+ (43%)	0.30 (7.00)	0.20 (−2.60)	0.25 (3.50)	0.09 (1.50)	0.36 (0.14)	0.32 (5.20)
R−S− (23%)	0.35 (7.50)	0.10 (1.40)	**0.66** (7.90)	0.06 (1.20)	**1.00** (0.34)	**0.98** (12.60)

Chronic conditions (32%)	0.32 (7.30)	0.25 (−3.40)	0.32 (4.70)	0.08 (1.50)	**0.51** (0.20)	**0.82** (11.60)
Healthy (68%)	0.16 (3.70)	0.04 (0.60)	0.35 (4.30)	0.01 (0.20)	**0.55** (0.19)	**0.68** (10.00)

Strong effects were highlighted (bold).

^∗^R+S− was not depicted (5%).
